# Assessment of Mechanical and Ecotoxicological Properties of Mortar with Wood Waste Biochar as Partial Cement Replacement

**DOI:** 10.3390/ma18040876

**Published:** 2025-02-17

**Authors:** Maša Legan, Petra Štukovnik, Klementina Zupan, Andreja Žgajnar Gotvajn

**Affiliations:** 1 Faculty of Chemistry and Chemical Technology, University of Ljubljana, Večna pot 113, 1000 Ljubljana, Slovenia; klementina.zupan@fkkt.uni-lj.si (K.Z.); andreja.zgajnar@fkkt.uni-lj.si (A.Ž.G.); 2 Faculty of Civil and Geodetic Engineering, University of Ljubljana, Jamova 2, 1000 Ljubljana, Slovenia; petra.stukovnik@fgg.uni-lj.si

**Keywords:** biochar, carbon footprint reduction, ecotoxicological properties, fresh mortar properties, hardened mortar properties, leachates, partial cement replacement

## Abstract

For several decades, cement production has caused concerns about CO_2_ emissions. As the production of concrete has increased over the years, the fact that cement is its key component additionally raises a concern. By partially replacing cement with waste material such as biomass waste biochar, the reduction in waste and the reduction of CO_2_ emissions could be addressed at the same time but raises a concern about the ecotoxicological potential of biochar-containing cementitious composites. During their use, recycling and disposal of biochar-containing mortars could pose hazardous environmental impacts due to their exposure to rain and other environmental conditions. The aim of the study was to determine the early-age mechanical properties of mortars with 5%, 10%, and 15% biochar as partial cement replacement. The environmental impact of biochar-containing mortars in terms of carbon footprint reduction and ecotoxicological potential was addressed simultaneously. The biochar used was prepared from waste wood biomass as carpentry waste wood. Results showed that added biochar caused no significant changes in flowability and fresh density of fresh mortar mixture. The strength tests revealed mortars with 5% and 10% biochar had higher 3-day flexural strength, while only mortar with 5% biochar had higher 7- and 28-day compressive strength (4% and 6%) than the conventional mortar. The X-ray diffraction (XRD) analysis detected five main crystalline phases in biochar-containing mortars. SEM-EDS showed the strong embedment of biochar particles in cement paste. Ecotoxicological assessment based on acute toxicity tests with mortar leachates using duckweed and mustard seeds showed low toxicity of leachates with the highest inhibition values around 50%. The calculations of the total CO_2_-equivalent emissions for selected mortars revealed mortars with biochar as partial cement replacement had lower CO_2_-equivalent emissions than the conventional mortar and can contribute to carbon footprint reduction and at the same time to natural resource conservation.

## 1. Introduction

Demand for raw materials for industries and production processes is high and will continue to increase in different sectors, especially in the construction sector. One of the most commonly used building materials in this sector are mortar and concrete. The two key components for mortar production are sand and cement, while concrete is composed of cement, fine aggregate (sand), and coarse aggregate (gravel or crushed stone). All components depend on a limited amount of natural resources, dispersed around the world unequally. The concrete industry is known to be one of the major consumers of natural resources [1,2,3]. Due to the ever-increasing demand for concrete, there is locally already a shortage of natural resources (sand, gravel, limestone) in some parts of the world. Besides a shortage of natural resources, cement production is also considered one of the largest anthropogenic sources of carbon dioxide (CO_2_) emissions, a prevailing greenhouse gas (GHG) [2,3,4,5,6]. Shah et al. [7] reported that cement production is responsible for 9% of all CO_2_ emissions.

In order to reduce the environmental impact of cement production, research has recently been focused on the development of new materials and alternatives, such as waste materials from various industries for cement replacement in cementitious composites. Past studies reported the possibility of using different wastes (glass, ceramic, wood waste ash), which could be reused as partial cement replacement [8,9,10,11,12]. Furthermore, many studies have recently reported on the possibility of using biochar in building materials as an additive or partial cement replacement in cementitious composites (mortar and concrete) [13,14]. Biochar is primarily known as carbon-rich material with a porous structure and high specific surface area. The main feedstock for biochar production is biomass waste mainly from forestry and agriculture including animal wastes, food industry wastes, and sewage sludge from wastewater treatment [15,16]. Biochar is produced by pyrolysis of biomass waste, which at the same time prevents the natural decomposition of biomass waste at landfills or reduces the need for its thermal processing, which are both sources of GHG emissions [17,18]. Conducted studies found biochar as an effective soil amendment [19,20]. Additionally, biochar application in livestock farming as a dietary supplement and even for the mitigation of odorous emissions from animal manure was reported [21,22].

Several studies confirmed that biochar use in cementitious composites has a great potential to partially replace cement by improving mechanical, physical, and chemical properties and at the same time contribute to climate change mitigation [13,23,24]. The improvement of building material properties could contribute to better durability, which can enhance the sustainability of building materials and reduce the demand for concrete and mortar. In addition, while most of the aforementioned biochar applications indirectly contribute to climate change mitigation, it was found that biochar in building materials can directly capture CO_2_ in its porous structure and thus directly contribute to carbon footprint reduction in relation to climate change mitigation [14,23,24,25].

Since mortars and concrete are building materials that have a long lifespan and are exposed to various environmental conditions, various substances can be leached from the mortars and concrete into the environment. Small particles of construction material can also be released or washed out of larger surfaces of building materials, which can be transported throughout the ecosystem. They can come into contact with rain, surface waters, sea water, and soil to leach out different substances. Formed leachates can have a negative effect on the aquatic and terrestrial organisms within the ecosystem. As waste materials have been commonly used in building materials in recent years, this increases the environmental risk, which is why recently ecotoxicological assessments have become very important for the determination of the ecotoxicological potential of such new building materials [26,27].

Additionally, wood is known to be a valuable natural resource for various purposes. While wood waste for biochar production often presents wastes from forestry, saw dust and wood waste from carpentry are also often used as feedstock. Wood for indoor and outdoor applications are often impregnated with wood preservatives for durability improvement, which can contain heavy metals [28,29]. During the formation of biochar through pyrolysis, some of these components are destroyed while some of them stay entrapped in biochar. At the same time, various pyrolysis products such as PAHs could be formed and adsorbed to biochar. This raises an additional concern about using wood waste biochar in cementitious composites in terms of the ecotoxicological potential of such materials [28,29].

While most of the studies about biochar as partial cement replacement in cementitious materials highlighted material properties by finding the proper content of biochar in composites, less attention was given to the ecotoxicological properties of such material during its production, use and end-of-life management, and assessment of carbon footprint reduction potential in relation to climate change mitigation. Therefore, it was considered to conduct a study to obtain these crucial data and to determine whether biochar in mortar can successfully partially replace cement without the deterioration of the material properties.

The purposes of this study were (1) to determine the early-age mechanical properties of cement mortars containing biochar as partial cement replacement; (2) to determine the ecotoxicological properties of biochar-containing mortar; and (3) to provide details on whether the use of such building materials improves carbon footprint reduction potential compared to mortar without biochar added.

## 2. Materials and Methods

### 2.1. Cement, Natural Aggregate, and Biochar

Ordinary Portland cement CEM I 42.5 N (Alpacem, Slovenia) conforming to Slovenian standard SIST EN 197-1:2011 [30] was used for this study. The cement was sealed and stored before its use in mortars. The density of Portland cement was 3110 kg m^−3^, and its Blain specific surface area was 3200 cm^2^ g^−1^. The chemical composition of cement CEM I is presented in Table 1. The loss on ignition (LOI) of the cement CEM I was 3.5%.

The ordinary natural aggregate (i.e., sand) was purchased from the quarry in Slovenia. Sand particles were sharp, unpolished, and unwashed. The maximum size of sand particles used for cement mortars was 4 mm. The water absorption of sand was 0.4% by mass, its density was 2700 kg m^−3^. The vast majority of the aggregate was calcite, while dolomite represented a very small part of the mineral content.

Beechwood waste from carpentry was used for biochar production. Wood waste was pyrolyzed in a muffle furnace at 700 °C in the absence of oxygen with a heating rate of 10 °C min^−1^ and residence time of 2 h. After the process of pyrolysis, the biochar was grounded and then sealed and stored before its application in cement mortars. A density of biochar (1280 kg m^−3^) was determined using gas pycnometer (Ultrapyc 5000, Anton Paar, Graz, Austria).

An elemental composition of biochar was determined using CHNS/O elemental analyzer (Perkin Elmer 2400 Series II, San Diego MA, USA) and is presented in Table 2.

The particle size distribution of biochar and cement was obtained using the particle size analyzer Microtrac S3500 Bluewave (Microtrac, Haan, Germany). The particle size distribution of the biochar and cement was determined in humid conditions, in which ethanol was used as a dispersant.

### 2.2. Fresh Mortar Properties

Biochar was added at 0, 5, 10, and 15% replacement by volume of cement in cement mortar (Table 2). Conventional cement mortar without biochar was used as the control mortar mix. A water to cement ratio of 0.45 was used in all of the cases in this study. Three samples of 40 mm × 40 mm × 160 mm were designed for each mortar mix. Superplasticizer Hiperplast 481 (TKK, Srpenica, Slovenia) was applied for all cement mortars. Its dosage was 0.7% of cement weight in conventional mortar. Design of cement mortars is presented in Table 3.

The flowability of fresh cement mortar mixtures was measured using a flow table test according to SIST EN 1015-3:2001 [31]. The test was performed by using the standard flow table on which the mold was first filled in two layers of mortar. Each layer was tamped 10 times with the tamper, then the mold was raised vertically and after 15 times of jolting of the table, two perpendicular spread diameters of the fresh mortar on the flow table were measured and the average of these two values presented the diameter of the fresh mortar mixture. Each mortar mix design consisted of 4 equal fresh mixtures of a volume of 2 L. The flowability of the mortar mix design was determined as the mean of the four aforementioned diameters.

The fresh density of cement mortars was determined by SIST EN 1015-6:1999 [32] by using a small container, which had a volume of approximately 1 dm^3^. Before the container was filled with fresh mortar mix, its weight was determined. The container was then filled with mortar until it was overflowing. The edges of the container were wiped clean and then the filled container was weighed. Each mortar mix design consisted of 4 equal fresh mixtures of a volume of 2 L. The fresh density of each mortar mix design was determined as the mean of the four measured weights.

### 2.3. Hardened Mortar Properties

The compressive and flexural strength of the prism samples were tested over a curing period of 3, 7, and 28 days. The prism samples were prepared by filling prism-shaped molds (40 mm × 40 mm × 160 mm) with mortars mixes (Table 2). A 3-point loading method was used on 3 parallel prism samples for the flexural strength test for each mortar mix. The flexural strength of each mix design was determined as the mean of the measured breaking load of 3 prism samples. Compressive strength was determined on the same samples used in the flexural strength test that split each prism into 2 parts, which were then used in the compressive strength test (6 samples). The compressive strength of each mix design was determined as the mean of the measured breaking load and cross-section area of the prism on which the load was applied (6 samples). Both strength tests were conforming to SIST EN 1015-11:2020 [33], by using a 50 kN testing machine for flexural strength and 500 kN for testing the compressive strength of the prism samples.

A PANalytical X’Pert PRO diffractometer was used for X-ray diffraction (XRD) analysis. XRD analysis was used for selected mortar samples to determine the crystalline hydration phases. Topas software version 2.1 was used for the quantitative analysis of XRD patterns to determine the mineral composition of the conventional and biochar-containing mortar samples.

Scanning electron microscopy (SEM-EDS, ULTRA Plus, Zeiss, Oberkochen, Germany) was used together with energy dispersive spectroscopy for detailed analysis of the surface morphology of mortars. Rietveld analysis was made with Topas software for the quantitative analysis of the XRD patterns of the selected mortars.

### 2.4. Ecotoxicological Assessment

#### 2.4.1. Preparation and Characterization of Leachates

Comparison of conventional and biochar-containing mortar mechanical properties revealed the most comparable biochar-containing mortar, i.e., B5 mortar, which was used for the ecotoxicological assessment. Thus, leachates for phytotoxicity tests were prepared from conventional and B5 mortar particles with diameters of less than 4 mm. Growth medium for duckweed (pH = 5.68 ± 0.01) and growth medium for mustard seeds (pH = 7.71 ± 0.02) were used for leachate production. Four different concentrations of mortar particles (100 mg L^−1^, 1 g L^−1^, 10 g L^−1^, and 100 g L^−1^) were used for leachates preparation in 500 mL Erlenmeyer flasks. The duckweed growth medium was prepared according to ISO guideline 20079:2005 [34] and was used for the duckweed test. The growth medium for the mustard seed germination test contained 11.76 g L^−1^ of CaCl_2 ▪_ 2H_2_O, 4.93 g L^−1^ of MgSO_4_, 2.59 g L^−1^ of NaHCO_3_, and 0.23 g L^−1^ of KCl [35,36]. Details about mortar leachate preparation are presented in Table 4.

Flasks containing growth medium and weighted mortar particles were covered with parafilm and placed on a laboratory shaker for 7 days (24 ± 2 °C; 150 rpm) to simulate the leaching process in the environment. According to the leaching test described in the SIST EN 12457-2:2004 [37] standard procedure, leachates with concentrations of 100 g L^−1^ were removed from the shaker after 24 h. All leachates were filtered using black ribbon and pH was measured (LE pH Electrode, FiveEasy F20, Mettler Toledo, OH, USA) after being removed from the shaker. The control sample (blank sample) presented only the growth medium for duckweed or mustard seeds and was treated the same as all mortar leachates. Prior to toxicity testing, the pH values of all the leachates were adjusted to 7.00 ± 0.10 due to their high alkaline nature with pH values up to 12.00. The pH of the control sample was also adjusted to 7.00 ± 0.10 using HCl and NaOH in accordance with SIST EN 12457-2:2004 [37] and ISO guideline 20079:2005 [34].

Leachates with the highest mortar concentration of 100 g L^−1^ were used for Inductively Coupled Plasma Optical Emission Spectroscopy (ICP-OES, Agilent 5100, CA, USA) as the highest concentration of leached substances was expected in these leachates. ICP-OES spectrometer (Agilent 5100 with detection limit of elements 10 ppb–10000 ppm) was used to determine the content of certain elements in selected leachates. Before the analysis, leachates were filtered and then acidified with 5% HNO_3_.

TOC analyzer Analytik Jena multi N/C 3100 was used to determine the possible total organic carbon (TOC) in the leachates.

Rapid tube tests (Nanocolor) were used for the determination of nitrite, nitrate, phosphate, sulfate, and chloride content in the leachate samples using Spectrophotometer Nanocolor VIS II, MACKEREI-NAGEL. Each leachate sample was tested twice.

#### 2.4.2. Phytotoxicity Tests

The duckweed phytotoxicity test using test plant Lemna minor was performed according to ISO 20079:2005 [34]. We used 100 mL beakers that contained 50 mL of the leachate/control. Each leachate concentration was performed in three parallel samples (replicates). In each beaker with the sample/control, 10 duckweed fronds were placed and, before this, the roots on the fronds were carefully removed. The beakers were then covered with parafilm and kept at a temperature of 24 ± 2 °C and exposed to a light cycle (16 h light/8 h dark) for 7 days. First, the frond number was scored and the growth rate in each leachate sample/control was determined after 7 day exposure. After growth rate determination, the root length of the duckweed fronds was measured using 1 mm graph paper.

For the determination of the total chlorophyll content, extraction in 95% ethanol was conducted. The prepared extracts were kept at a temperature of −18 ± 2 °C in complete dark for 24 h. The absorbance of the samples at 664.2 nm (chlorophyll a) and 648.6 nm (chlorophyll b) wavelength values were determined after 24 h, using a multimode microplate reader (BioTek Synergy LX, Agilent, CA, USA). Concentrations of chlorophyll a and b were determined according to equations presented by Lichtenthaler [38]. The total chlorophyll content was defined as the sum of the contents of chlorophyll a and chlorophyll b. The inhibition of the frond number and root growth in the mortar leachates was determined as a comparison to the values in the control sample.

The mustard seed germination phytotoxicity test was performed in covered Petri dishes. Smaller circular filter paper was put on the bottom of the Petri dish and 3.5 mL of the leachate/control was added. After filter paper soaked all the leachate/control sample, 20 mustard seeds were placed on it in 4 lines with 5 seeds. The Petri dishes were then covered with glass Petri dish covers and put inside the incubator for 72 h at a temperature of 21 ± 1 °C in total absence of light. For each leachate/control sample, 2 parallel (replicates) samples were prepared. After 72 h, the root length of the mustards seeds was measured. The inhibition of mustard seed root growth in the mortar leachates was determined by comparison to the values in the control sample.

## 3. Results and Discussion

### 3.1. Flowability and Fresh Density

The flow diameters and fresh densities are presented in Table 4. Results of the flow table test showed no significant changes in the flowability of fresh mortar mixes due to the presence of biochar in the mortar mix. It was found that measured flow diameters of biochar-containing mortars were comparable to the control with a flow diameter of 127 mm. The fresh densities of biochar-containing mortars and conventional mortar are presented in Table 5. It was noticed that fresh density mortars slightly increased (from 2229.35 to 2337.35 kg m^−3^) with the increasing content of biochar (5–15%), but the overall comparison to conventional mortar showed insignificant changes. The same results were found in the study conducted by Maljaee et al. [39] in which biochar content had no significant effect on the fresh density of mortar. In addition, Gupta et al. [40] noticed the fresh density of biochar-containing mortar mixes decreased with increasing content of biochar and attributed to the porous structure and lower density of mortar.

### 3.2. Particle Size Distribution of Biochar and Cement

**Figure 1:**
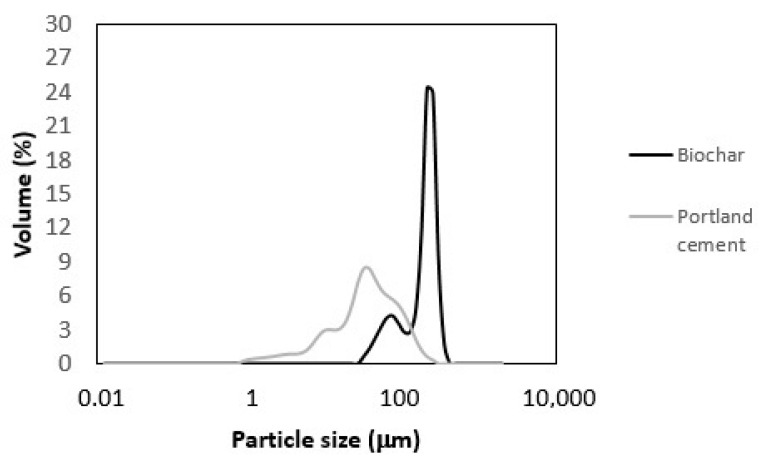
Particle size distribution of biochar and Portland cement.

The particle size distribution of biochar and cement is shown in Figure 1. Both particle size distributions were determined by the same method in humid conditions. Results showed that the particle sizes of biochar varied between 30 and 350 µm, while the biggest part of almost 60% of the total volume presented particles was in the range of 150 µm to 250 µm. The particle sizes of Portland cement ranged from 0.8 µm to 250 µm, where 80% of particles were smaller than 100 µm. A comparison of biochar and Portland cement particle size distribution showed that cement particles were, in general, smaller than biochar particles. On the contrary, Gupta et al. [40] and Qin et al. [41] reported that biochar particles were finer than cement. However, Tan et al. [42] found in their review that the particle size of biochar depends on the grinding method and on the duration of the grinding process, which is the main reason for such differences in the particle size of biochar.

### 3.3. Flexural Strength

**Figure 2:**
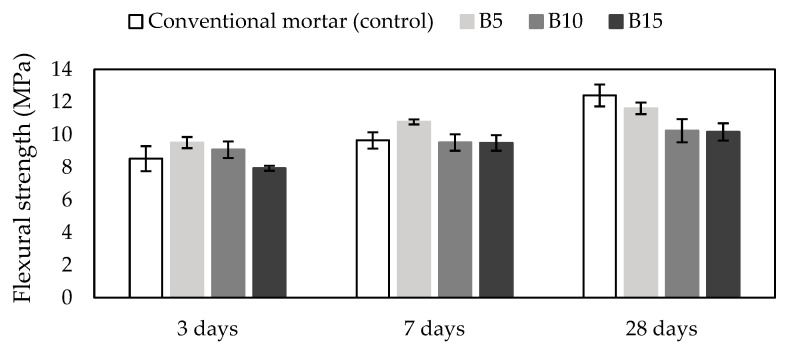
Flexural strength of mortars. Notes: B5 = 5% biochar; B10 = 10% biochar; B15 = 15% biochar.

The flexural strength of biochar-containing mortars and conventional mortar as the control are shown in Figure 2. The 3-day flexural strength of B15 mortar with 15% biochar as cement replacement was lower by 7% than the control, while the strength of the B5 and B10 mortars increased by 11% and 6% compared to the control (8.5 MPa). The comparison of 7-day strength showed that B5 mortar had the highest strength of 10.8 MPa among all tested mortars. Also, B10 and B15 had lower strength than the control, but those differences were around 1% (0.15 MPa). Interestingly, an analysis of 28-day flexural strength results revealed that all of the strengths of the mortars with biochar content were lower than the strength of conventional mortar (12.4 MPa). It was found that the flexural strength of B5, B10, and B15 was 94%, 83%, and 82%, respectively, of that of the control cement mortar.

Maljaee et al. [39] conducted a study to find the optimal cement replacement in which different types of biochar were used as cement replacement in mortars. It was found that biochar content in mortar differently influenced 7- and 28-day flexural strength. The 28-day flexural strength of mortars with biochar content of 0.5%, 1%, 2%, and 4% showed an increase compared to conventional mortar [39]. In contrast, the results of our study showed completely opposite findings than aforementioned study. It can be assumed that the biochar content of more than 4% decreases flexural strength and 4% biochar as cement replacement represents the optimal dosage of biochar in mortar without flexural strength deterioration. Since 5%, 10%, and 15% in our study exceed this optimal dosage of biochar, the flexural strength in our study decreased compared to conventional mortar.

Additionally, Gupta et al. [40] found that 28-day flexural strength of cement mortars with biochar from different biomass waste (mixed wood sawdust) as additive in contents of 1%, 2%, and 5% did not significantly influence flexural strength. However, one mortar mix with a biochar content of 1% by weight of cement in mortar showed an increase up to 5% compared to the control. An increase in 3- and 7-day flexural strength of the B5 mortar was also determined compared to the control, while 28-day flexural strength decreased (5%) in comparison to conventional mortar. Ahmad et al. [43] reported on the possibility that the decrease in flexural strength of biochar-containing cement mortars is linked to a very large number of small biochar particles, which may be attributed to a large number of inert inclusions in the mortar matrix. These could also explain a decrease in 28-day flexural strength in our study for all of the biochar-containing mortars, regardless of the biochar content.

### 3.4. Compressive Strength

**Figure 3:**
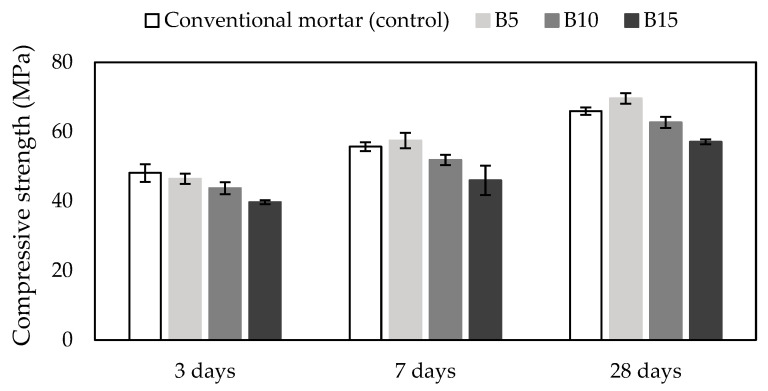
Compressive strength of mortars. Notes: B5 = 5% biochar; B10 = 10% biochar; B15 = 15% biochar.

The 3-, 7-, and 28-day compressive strength of conventional cement mortar as the control and biochar-containing cement mortars mixes are presented in Figure 3. The comparison of compressive strength of 3-day mortars indicated that all of the biochar-containing mortars showed a decrease in compressive strength compared to conventional mortar. The most significant decrease of 21% was the B15 mortar, while the B5 mortar had the smallest decrease (almost 4%). The 7-day compressive strengths showed that the B5 mortar had the highest strength of 57.5 MPa, while the control had a strength of 55.7 MPa. The biochar-containing mortars B10 and B15 had lower compressive strength than the control (6% and 17%). The 28-day compressive strength of the B5 mortar showed even better improvement compared to the 28-day strength of conventional mortar. Despite improvements, only the B5 mortar strength of 69.6 MPa was higher by almost 6% than the conventional mortar strength (65.9 MPa). The most significant increase in compressive strength through the period of 28 days showed mortar B5 containing 5% biochar with an improvement of strength by 33%. Overall, 10% and 15% biochar from wood waste as cement replacement in mortars reduced compressive strength (4% and 13%), while 5% biochar in mortar increased it up to 6% compared to conventional mortar and increased further with the age of the mortar sample.

Roychand et al. [44] used biosolids, bioash, and biochar as 5 and 10% cement replacement. Similar results were reported on compressive strength reduction up to 9% in comparison to the control mortar mix when cement was partially replaced with 5% and 10% biochar. It was also found that an increase in biochar content in the mortar up to 10% only added to the reduction in compressive strength. Among all materials used as cement replacement, biochar was labeled as the most appropriate one [44]. According to the results of our study, 5% biochar increased compressive strength, which is contrary to the results reported by Roychand et al. [44]. Based on particle size distribution (Figure 1), almost 60% of the total volume of biochar particles in our study was in the range of 150–250 µm, while Roychand et al. [44] reported 50% of the total volume of biochar presented particles with diameters up to 13 μm. Therefore, it can be assumed that when replacing cement, a smaller diameter of biochar particles can contribute to a decrease in compressive strength.

The use of biochar from different biomass waste (food and wood waste) as an additive in the mortar was studied by Gupta et al. [45]. The results presented in the study showed that the addition of 1%, 2%, and 5% biochar by weight of cement after 7 and 28 days increased compressive strength up to 20% compared to conventional mortar, while 5% biochar from food waste and rice waste as cement replacement reduced it up to 15%. It was concluded that the optimal amount of biochar as an additive in mortar is 1% [45], which is similar to the optimal amount of biochar as cement replacement in mortar reported by Maljaee et al. [39]. However, according to the results of our study, 5% biochar as cement replacement increased 7- and 28-day compressive strength compared to conventional mortar. This indicates that the optimal content of biochar for compressive strength improvement could be 5% when replacing cement.

Choi et al. [46] used hardwood biochar as 5, 10, 15, and 20% cement replacement in mortar. It was found that 28-day compressive strength decreased with an increase in biochar content, while the sample with 5% biochar presented an exception with an increase up to 10% compared to the control [38]. These results are in accordance with the increase of 28-day compressive strength of the B5 mortar in our study.

Furthermore, Gupta and Kua [47] reported that the addition of biochar in mortar lowers the water-to-cement ratio because the particles of biochar absorb water during the mixing process, which contributes to the densification of hardened mortar. Also, the absorbed water in biochar particles is later during the process of hardening of mortar supplied to the mortar paste where biochar plays the role of an internal self-curing agent that contributes to the development of strength [46,47]. Several past studies suggested that the presence of biochar in mortar as cement replacement generates a filler effect [47,48]. This contributes to the increase in the strength of the mortar. Based on the results of our study about early-age strength (3- and 7-day strength), the filler effect due to the biochar presence in the composite led to an increase in early-age flexural and compressive strength compared to conventional mortar. It can be assumed that the filler effect is attributed to a reduction in the water-to-cement ratio around biochar particles in the mortar mix and then to an improvement of hydration (higher content of hydration products) due to the nucleation effect of biochar particles that provide an adequate surface for the precipitation of cement hydration products.

One of the few studies, which focused on the early strength of cement mortars with different biochar contents (3% and 1%), was conducted by Gupta and Kashani [49]. Results of the study showed that the 1- and 3-day compressive strength of unwashed peanut shell biochar-containing mortars increased by almost 40% compared to the conventional mortar (control). Also, the mortar with a biochar content of 3% had lower strength compared to the mortar with a biochar content of 1%. It can be concluded that those differences in early compressive strength arise because biochar in cement mortars induces a so-called filler effect that has a positive effect on the hydration process, which results in early compressive strength improvement [49].

### 3.5. XRD of Mortar Samples

**Figure 4:**
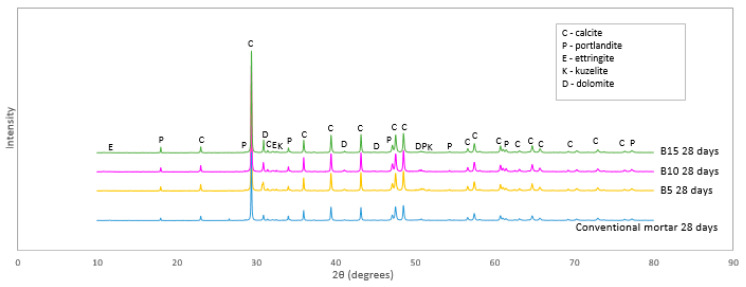
XRD patterns.

XRD was conducted to identify the main hydration phases formed in cement mortars due to the addition of biochar. According to the compressive and flexural strength presented in Figure 2 and Figure 3, it was found that the most significant changes in strength were observed in investigated mortars at 3 and 7 days of curing. To obtain more details about these changes in the samples, 3- and 7-day B5, B10, and B15 were used for XRD analysis. The diffractogram of the selected mortars is presented in Figure 4.

From the XRD patterns of biochar-containing mortars, five main crystalline phases were detected: calcite (C), dolomite (D), portlandite (P), ettringite (E), and kuzelite (K). The peaks of calcite and dolomite occurred due to the presence of natural aggregate, which mostly contained calcite and a significantly lower amount of dolomite. Among all selected mortars, the B5 and B10 mortars had the most intense peaks of calcite and portlandite while B15’s intensities were the lowest. The most intense peaks presented calcite, while the lowest intensity peaks belonged to kuzelite. Ettringite intensity peaks were also detected of which the highest ones were noticed for the conventional and B5 mortars. Overall, the highest intensities of crystalline phases were presented by the conventional and B5 mortar samples, which were comparable and can, therefore, confirm the similarity of 28-day compressive and flexural strength of the B5 and conventional mortars.

The comparison of XRD patterns of biochar-containing mortars confirmed that the increasing content of biochar in mortars reduced the peak intensities of crystalline phases. Furthermore, it was noticed that the increased content of biochar in mortars did not significantly change the position (2θ) of crystalline compounds inside cement mortars.

Quantitative analysis of the XRD patterns revealed differences in mineral composition of the mortar samples (Table 6). It was expected that increasing biochar content led to lower content of cement hydration products. However, the comparison of results showed ettringite content was the highest in the B5 mortar (2.43 ± 0.51%), while the conventional mortar had more than 2 times lower ettringite content. According to Zhang et al. [50], it can be assumed that biochar promoted and prolonged the hydration process, contributing to the formation of a higher content of hydration products such as ettringite. While biochar contents of 10% and 15% showed a decrease in ettringite content, 5% biochar as cement replacement in our study presented an optimal content of biochar with no deterioration of mechanical properties.

Maljaee et al. [39] reported similar results of reduced peak intensities of crystalline phases due to an increase in biochar content in the mortar. It was also noticed that the biochar addition improved the hydration process by promoting hydration products inside cement mortar compared to plain cement [39]. Gupta et al. [40] indicated that there is a possibility that absorbed water in biochar pores presents the appropriate conditions for the formation of hydration products. Also, Qin et al. [41] investigated the addition of biochar in previous concrete and highlighted that when biochar addition in concrete was more than 6.5% by weight of cement, a large number of biochar particles in the composite can generate the agglomeration effect. Furthermore, the agglomeration of biochar particles in the composite can therefore cause the creation of localized weak zones [40,45].

### 3.6. SEM

**Figure 5:**
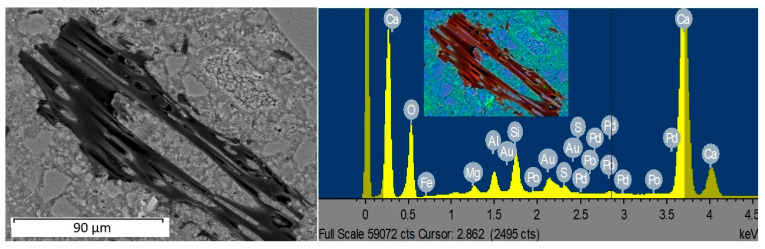
SEM-EDS image. Notes: Red color = carbon (biochar); blue color = natural aggregate; green color = cement paste.

The surface morphology of the conventional mortar and B5 biochar-containing mortar was studied by SEM and EDS. SEM-EDS images are presented in Figure 5. After surface examination of the samples, it can be visualized that biochar particles were strongly embedded in hardened cement paste as aggregate particles in the mortar samples. In some of the past studies, products of the hydration process were found in the pores of biochar [40,45,51], which probably contributed to the improvement of early strength compared to the conventional mortar in our study.

### 3.7. Phytotoxicity Tests with Duckweed and Mustard Seeds

**Figure 6:**
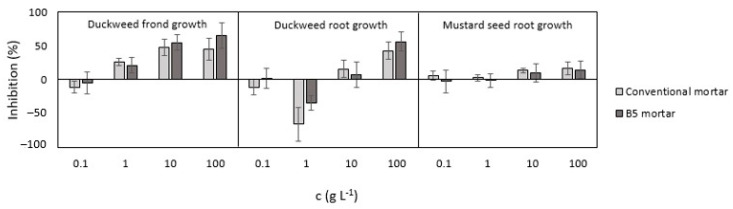
Phytotoxicity test results.

The analysis of the 7-day duckweed test indicated that the inhibition of frond growth increases with increasing leachate concentration (Figure 6). The 100 mg L^−1^, 1 g L^−1^, and 10 g L^−1^ leachates of the conventional and B5 mortars showed comparable inhibition values. While the 100 mg L^−1^ leachates of the conventional and B5 mortars promoted frond growth (−11% and −5%), the 1 g L^−1^ and 10 g L^−1^ leachates inhibited duckweed frond growth. The inhibition of the 1 g L^−1^ B5 mortar was 20% lower than the conventional mortar leachate (26%), while the 10 g L^−1^ B5 mortar’s inhibition was 13% higher than the leachate of the conventional mortar (48%). Leachates with the highest concentration of mortar (100 g L^−1^) showed that frond number inhibition of the B5 mortar leachate (65%) was significantly higher than the inhibition of the conventional mortar (45%). According to the large standard deviations presented in Figure 6, it can be seen that the inhibition values in the 10 g L^−1^ and 100 g L^−1^ leachates were comparable. It can also be assumed that due to the inhomogeneous structure of hardened mortars, different amounts of cement, natural aggregate, and biochar could appear in the weighted amount of mortars for leachate preparation. Therefore, large deviation appeared in the 100 mg L^−1^ and 100 g L^−1^ mortar leachates. However, based on the results of our study, the inhibition values indicated that both mortars as non-toxic.

Root growth inhibition showed that the 100 g L^−1^ leachates had the highest inhibition (43% and 56%) compared to the control. The B5 100 g L^−1^ mortar leachate had a higher toxic effect to the root growth of duckweed than the conventional mortar leachate. It was also found that the 1 g L^−1^ of conventional and B5 mortar leachates promoted root growth significantly (−66% and −34%). The 100 mg L^−1^ of conventional mortar leachate showed slight promotion (-11%), and the B5 mortar leachate inhibited duckweed root growth (2%). Large standard deviations, which were also noticed in duckweed frond growth inhibition, were also noticed for root growth. Therefore, the comparison of the 100 mg L^−1^ and 10 g L^−1^ mortar leachate results and considering the data distribution (standard deviation) for both mortars revealed their comparability. Only the leachate of the 10 g L^−1^ B5 mortar showed less toxic effect (7% inhibition) than the conventional mortar leachate (16%), while other biochar-containing mortar leachates showed more toxic effect than the conventional mortar. Despite the aforementioned, both mortars could be labeled as non-toxic to an aquatic environment. The inhomogeneity of the grinded mortar samples for leachate preparation could play an even more important role than in duckweed frond growth. It can be assumed that prepared leachates differentiate in biochar composition, natural aggregate, and cement paste content, which can therefore affect standard deviations.

**Figure 7:**
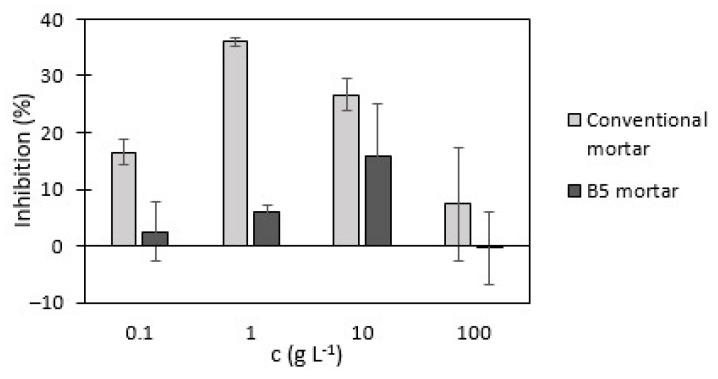
Inhibition of total chlorophyll content in duckweed.

According to the inhibition of total chlorophyll content in duckweed presented in Figure 7, all biochar-containing mortar leachates had significantly lower inhibition values than conventional mortar leachates. The highest inhibition value of total chlorophyll content was caused by the 1 g L^−1^ conventional mortar leachate (36%), while the highest inhibition in biochar-containing mortar leachates was in the 10 g L^−1^ leachate (15%). Comparison of the 100 mg L^−1^ and 100 g L^−1^ inhibition revealed that leachates prepared with the lowest amount of the conventional and B5 mortars contributed to higher inhibition values (16% and 2%). The 100 g L^−1^ conventional and B5 mortar leachates had lower values (7% and −0.3%). It can be assumed that the difference in inhibition between the highest and the lowest mortar leachate concentrations was related to leaching time. According to SIST EN 12457-2:2004 [37], leachates with concentrations of 100 g L^−1^ were removed from the shaker and filtered after 24 h, while the 100 mg L^−1^ leachates were removed and filtered after 7 days. Despite the higher amount of mortar in the 100 g L^−1^ leachate, fewer substances leached out of the mortar particles and affected total chlorophyll content. No such differences were noticed in the frond growth and root growth inhibition results.

In one of the few studies that addressed ecotoxicity of concrete conducted by Mocova et al. [52], a 100% inhibition of frond growth and total chlorophyll content was found in all conventional and recycled concrete leachates regardless of concentration and with no pH adjustment. The pH values were up to 12.3. After pH adjustment to pH value of 7.0, it was concluded that recycled concrete leachates slightly promote duckweed frond growth compared to conventional concrete leachates. Also, the results of total chlorophyll content inhibition showed that conventional concrete leachates promoted chlorophyll content up to 20%, while recycled concrete leachate inhibited chlorophyll content up to 30% regardless of the concrete leachate concentration rate (6.25, 12.5, 25, 50, and 100%). According to the inhibition results of our study, where pH adjustment of leachate was also made, more significant changes between different concentrations of leachate were noticed, especially biochar-containing mortar leachates.

The importance of feedstock for biochar production and ecotoxicology assessment of four different biochars was presented by Oleszczuk et al. [53]. The ecotoxicity of biochars was performed using plant *Lepidium sativum* (garden cress). Biochars from elephant grass, wicker, coconut shell, and wheat straw were used for leachate preparation. Three different dosages (1%, 5%, 10%) of each biochar were added to OECD soil and after 3 days, the length of the *Lepidium sativum* was measured. It was found that 1% biochar in soil promotes root growth with inhibition values from −1% to −40%, while 5% and 10% biochar added inhibited root growth up to 92%. The highest inhibition caused biochar from elephant grass. After further analysis of biochar, it was also found that the elephant grass biochar contained the highest amounts of heavy metals, which could contribute to these significant toxic effects. Biochar leachates were also prepared according to SIST EN 12457-2:2004 [37], using biochar and deionized water (1 L of water/100 g of biochar). Toxicity of the leachates was determined using four additional test organisms: bacteria (*Vibrio fischeri*), algae (*Selenastrum capricornutum*), protozoa (*Tetrahymena therophila*), and crustaceans (*Daphnia magna*). It was concluded that *Daphnia magna* was the most sensitive organism. The toxic impact of polycyclic aromatic hydrocarbon (PAH) content in biochar was also noticed only in the *Daphnia magna* test [45]. This indicated, not only the heavy metal content, but also that the content of PAHs in biochar could cause a toxic effect on different aquatic and terrestrial organisms.

The results of the mustard seed germination test presented in Figure 6 revealed that all biochar-containing mortar leachate had lower inhibition values of root growth than conventional mortar leachates. The 10 g L^−1^ and 100 g L^−1^ conventional and B5 mortar leachates showed similar inhibition of root growth. The highest inhibition of root growth was in the 100 g L^−1^ conventional mortar leachate (17%). Comparison of the 10 g L^−1^ and 100 g L^−1^ conventional and B5 mortar leachate inhibitions showed that the biochar-containing mortar leachate had up to 23% lower inhibition. According to the 100 mg L^−1^ and 1 g L^−1^ B5 and conventional mortar leachate inhibition, both showed similar inhibition values of mustard seed root growth from −3% to 5%. Large deviations were found in biochar-containing mortar leachates, which could indicate the differences in biochar content in biochar-containing mortars. This indicates that the inhomogeneity of biochar-containing mortar, especially due to biochar content, contributed to such data distribution and, therefore, determined the inhibition in our study. Based on inhibition, both mortars could be labeled as non-toxic.

Stefaniuk et al. [54] investigated the toxic effects of different biochars on *Lepidium sativum*. Biochars were residues made from biogas production and were added to OECD soil (at rates 0.5% and 5%) and the biochar leachates were prepared for ecotoxicological assessment. It was found that the most negative impact of biochar on test plant root growth was related to the presence of both PAHs and heavy metals (Cr, Cu, Cd, Pb, and Mn) in biochar samples. Additionally, pyrolysis temperature was also found to be one of the most important parameters during biochar production. It was concluded that high temperature (800 °C) contributed to an increase in the content of PAHs and heavy metals (Cr, Cu, Cd, Pb, and Mn), which increase the toxic effect of biochars on test plants. In our study, pyrolysis temperature of 700 °C with a heating rate of 10 °C min^−1^ and residence time of 2 h could, therefore, also contribute to the inhibition of mustard seed root growth due to the potential of PAH formation.

### 3.8. Characterization of Mortar Leachates

The concentrations of nutrients in the conventional and biochar-containing mortar leachates are presented in Table 7. 

A comparison of the duckweed growth medium with no mortar content, which served as control, and both mortar leachates showed no significant difference in nitrite content. The conventional mortar leachate contained 10% less nitrate than the B5 mortar leachate, while both mortar leachates had 99% lower concentrations of phosphate than growth medium. Such differences in phosphate could be explained based on the results of the study conducted by Liu et al. [55], in which Portland cement was used as an effective material for total phosphorus concentration reduction in eutrophic water. It was reported that only a small amount of cement (up to 4 g L^−1^ of eutrophic water) could remove up to 90% of phosphate and increase the acidic pH values of such water to 12. Regarding Walsh et al. [56], an optimal concentration of phosphate is 0.1–90.2 mg L^−1^ for duckweed. According to the concentrations in mortar leachates in our study, the phosphate concentration is within this optimal range. A sulfate concentration in mortar leachates was up to 29% lower than in the growth medium, while chloride concentration significantly increased in the conventional and B5 mortar leachates (4.1 mg L^−1^ and 7.2 mg L^−1^). According to the optimal chloride concentration range of 0.03–199.94 mg L^−1^ [56], the chloride concentration in mortar leachates was within this range and could not cause a significant toxic effect on duckweed.

The results of the ICP-OES analysis are presented in Table 8. In both mortar leachates, the highest content presented calcium (Ca), silica (Si), and barium (Ba). The main sources of Ca, Si, and Ba are cement and natural aggregate. Their presence mostly contributes to the alkalinity of mortar leachates (pH > 7). Walsh et al. [56] reported that high Ca content could contribute to seed germination inhibition. An optimal concentration range of 8–800 mg L^−1^ for duckweed was also reported [48]. Regardless of the 4 times higher Ca concentration in the mortar leachates compared to the growth medium, all concentrations were within the optimal concentration range. Since Si is an important element in the ecosystems, its increased content in mortar leachates does not raise concern regarding its toxic effect on duckweed. In contrast, the increased Ba content in mortar leachates raises concern in relation to its toxic impact on duckweed according to Wang [57], but some research still needs to be conducted to confirm such findings.

According to some past studies about the ecotoxicological assessment of biochar, heavy metal and PAH contents could be the main cause for the toxic impact on different organisms. Based on the results of our study, the content of Cr increased in both mortar leachates. However, Cr content in the biochar-containing mortar was almost 2 times higher than in the conventional mortar leachate, which could be the result of biochar in mortar as partial cement replacement. Past studies reported that Cr concentrations in biochar ranged from 4 to 39 mg kg^−1^ biochar [53,54]. When preparing leachates, some of this Cr leached into the growth medium, which could be one of the causes for increased Cr content in our study and contributes to the increased toxic effect of leachates on duckweed.

Reale et al. [58] performed the duckweed growth inhibition test according to the OECD Guideline for the testing of chemicals No. 221. SIS growth medium was used for the preparation of five different concentrations of potassium dichromate K_2_Cr_2_O_7_ (0.50, 0.93, 1.73, 3.22, 6.00 mg L^−1^), which were expressed as concentrations of Cr 3.4, 6.3, 11.7, 21.9, and 40.7 μM solutions. After 7 days, results of the test revealed a decrease in the growth rate of duckweed in all solutions containing Cr compared to the control. The highest inhibition of duckweed growth rate (40%) was found in the solution with the highest concentration of Cr (40.7 μM), while the lowest Cr concentration (3.4 μM) inhibited the growth rate by 13% [58]. According to the Cr concentration in our study (9.4 μg equals to 0.17 μM), the Cr concentration was lower than the lowest investigated Cr concentration in the aforementioned study. Therefore, it can be concluded that Cr content in our biochar-containing leachate did not significantly contribute to duckweed frond number and root growth inhibition.

Aslanzadeh et al. [59] reported on the potential of Cr (VI) removal from wastewater using duckweed. K_2_Cr_2_O_7_ was dissolved in Hoagland medium with Cr (VI) concentrations of 1, 10, 50, and 100 mg L^−1^ at three different pH values (5, 7, and 9). It was found that duckweed in synthetic wastewater with 1 mg L^−1^ of Cr (VI) at a pH of 7 absorbed 75% of Cr (VI), while 10 mg L^−1^ Cr (VI) synthetic wastewater absorbed only 30% of Cr (IV). Also, the majority of Cr (VI) was absorbed in the first 24h of exposure. The efficiency of Cr (VI) removal in the first 24h ranged from 14 to 35%. According to the Cr concentration in biochar-containing mortar leachate in our study, it can be assumed that the majority of the Cr in leachate could be absorbed by duckweed. However, an analysis of biochar-containing leachate after the duckweed phytotoxicity test should be conducted to confirm efficient Cr removal.

Additionally, TOC measurements of both mortar leachates confirmed that there was no presence of organic compounds (less than 0.500 mg L^−1^).

### 3.9. Potential of Carbon Footprint Reduction

Several past studies reported on the carbon content in biochars produced from different types of biomass waste, while only a few used beech wood waste as feedstock for biochar production. In the study conducted by Wystalska and Kwarciak-Kozłowska [60], beech wood chips were used for biochar production (pyrolysis at 700 °C). The results showed a high carbon content of 84.6% compared to other biochars that were produced from walnut shells and wheat and rye straws [60].

In addition, Ibarrola et al. [61] made a comparison of carbon (equivalent) abatements of different biochar production processes from different biomass waste. It was found that slow pyrolysis systems were associated with the highest net carbon abatements especially for biochar production from wood waste (1.25 kg of CO_2_-eq kg^−1^ of biochar) [61]. This value was also used in the current study in which the net CO_2_ emission associated with the production of wood waste biochar is −1.25 kg of CO_2_ equivalents kg^−1^ of biochar [61], while Portland cement contributes 1.002 kg of CO_2_ kg^−1^ of Portland cement [62], and sand contributes 2.21 × 10^−3^ kg of CO_2_ kg^−1^ of sand [63]. These values are presented in Table 9.

Considering the high carbon content of beech wood biochar and its contribution to higher avoidance of certain amounts of CO_2_-equivalent emission compared to other biochars, the calculation for the total CO_2_-eq emissions of the conventional mortar and B5 mortar mix were made. The results of the total CO_2_-eq for selected mortars are presented in Table 10. It was found that 5% biochar as cement replacement in mortar reduced CO_2_-eq by 7.5% compared to conventional mortar. The main cause for this reduction is that biochar’s negative CO_2_-eq replaced the relatively high CO_2_-eq of Portland cement. These results indicate that biochar-containing mortars have great potential for carbon footprint reduction.

The main reason for the selection of the B5 mortar mix for the aforementioned calculations and comparison with conventional mortar was because strength tests and XRD analysis showed that B5 had comparable or better properties than conventional mortar. Improved early-age properties due to biochar addition contribute to the enhanced durability of mortar. Also, durability enhances the sustainability of building material (mortar and concrete) and prolongs its service life. This can reduce the demand for cement and natural aggregates as key ingredients for mortar and concrete, and at the same time contribute to climate change mitigation [64].

Additionally, in a recent review [23], it was reported that biochar-containing building materials can directly or indirectly contribute to carbon footprint reduction. The contribute indirectly by reusing waste biomass for biochar production, which contributes to biomass waste reduction instead of natural decomposition in landfills or incineration. The capability of biochar in building materials to directly capture CO_2_ from the air in its pores was highlighted as a direct contribution to carbon footprint reduction [25,65].

However, there is a need to find the optimal cement replacement rate that would at the same time improve the mechanical, chemical, and physical properties of cementitious materials and contribute to the reduction of the carbon footprint. This will most likely present the biggest challenge in the future in the area of biochar-containing building materials.

## 4. Conclusions

Determination of early-age mechanical and ecotoxicological properties of biochar-containing mortar showed the great potential of such material. The early-age mechanical properties of biochar-containing mortar were comparable to conventional mortar and improved in terms of compressive strength, when replacing 5% of cement with biochar. Moreover, 5% biochar in mortar increased 3- and 7-day flexural strength by 11%, while all biochar-containing mortars had lower 28-day flexural strengths than conventional mortar. However, the flexural strength of mortar with 5% biochar decreased only up to 7% compared to conventional mortar. The compressive strength results showed that 10% and 15% biochar in mortar decreased, while 5% biochar increased 7- and 28-day compressive strengths up to 4% and 6% compared to conventional mortar.

The ecotoxicological assessment of mortar and biochar-containing mortar leachates showed that both mortars could be labeled as non-toxic in aquatic and terrestrial environments. The duckweed test revealed the inhibition of frond growth and root growth in leachates prepared with the highest concentration of mortar (100 g L^−1^), which slightly exceeded 50%. According to the mustard seed germination test results, the inhibition values occurred regardless of the mortar leachate concentration lower than 20%. Despite the biochar content inside the mortar structure, the ecotoxicological assessment revealed no increased toxic effect on test organisms. When comparing both mortars, the determined mechanical and ecotoxicological properties confirmed biochar-containing mortar’s suitability and its great potential to successfully replace conventional mortar.

The calculations regarding carbon footprint reduction potential also showed that 5% biochar as cement replacement can reduce CO_2_-equivalent by 7.5% compared to conventional mortar. This additionally confirmed the great potential of the actual use of such materials in the future, although some research still needs to be conducted.

Some limitations may have impacted the main findings of this study, especially large standard deviations and data distribution, which resulted in lower repeatability. Heavy metal content also raises concern in biochar-containing mortar leachates due to the inhomogeneity of the mortar samples for leachate preparation. However, the inhomogeneity is also present in nature, when leaching of mortar could appear.

## Figures and Tables

**Table 1:** Chemical composition of Portland cement CEM I 42.5 N.

Oxide	CaO	SiO_2_	Al_2_O_3_	Fe_2_O_3_	SO_3_	MgO	K_2_O	Na_2_O	Cl	Others
Content (%)	63.49	19.68	4.82	2.88	2.74	1.54	0.72	0.34	0.06	0.23

**Table 2:** Elemental composition of biochar.

Element	C	H	N	S	O
Content (%)	87.29	0.43	0.57	0.01	11.70 *

Note: * = calculated by difference.

**Table 3:** Design of cement mortars.

Component	Content (kg m^−3^ of Cement Mortar)			
	Conventional Mortar	B5	B10	B15
Cement	450.00	427.50	405.00	382.50
Water	209.19	209.19	209.19	209.19
Sand	1673.62	1673.62	1673.62	1673.62
Biochar	/	9.29	18.58	27.87
Superplasticizer	9.45	9.45	9.45	9.45

Notes: B5 = 5% biochar; B10 = 10% biochar; B15 = 15% biochar.

**Table 4:** Mortar leachate preparation details.

	Duckweed Phytotoxicity Test					Mustard Seed Phytotoxicity Test				
Mortar leachate concentration (g L^−1^)	0	0.1	1	10	100	0	0.1	1	10	100
Mortar weight (g)	0	0.1	1	10	100	0	0.1	1	10	100
Leaching time (h)	168	168	168	168	24	168	168	168	168	24
Growth medium (composition)	ISO guideline 20079:2005 (Steinberg medium)					11.76 g L^−1^ of CaCl_2 ▪_ 2H_2_O, 4.93 g L^−1^ of MgSO_4_, 2.59 g L^−1^ of NaHCO_3_, 0.23 g L^−1^ of KCl				

**Table 5:** Flowability and fresh density of mortars.

	Mortar Mix			
	Conventional Mortar	B5	B10	B15
Flow diameter (mm)	127.0 ± 2.5	120.0 ± 1.2	126.2 ± 0.8	131.0 ± 0.7
Fresh density (kg m^−3^)	2342.10 ± 0.30	2291.35 ± 7.95	2320.50 ± 2.00	2337.35 ± 0.75

Notes: B5 = 5% biochar; B10 = 10% biochar; B15 = 15% biochar.

**Table 6:** Mineral composition of conventional and biochar-containing mortars.

	Mortar Mix			
Mineral Content (%)	Conventional Mortar	B5 Mortar	B10 Mortar	B15 Mortar
Calcite	85.75 ± 1.73	85.06 ± 1.26	82.70 ± 0.81	81.41 ± 1.02
Dolomite	6.78 ± 0.71	6.42 ± 1.03	9.90 ± 0.56	11.22 ± 1.61
Portlandite	5.26 ± 0.48	4.41 ± 0.62	4.38 ± 0.53	3.69 ± 0.57
Ettringite	1.07 ± 0.23	2.43 ± 0.51	0.91 ± 0.29	0.63 ± 0.28
Kuzelite	1.14 ± 0.15	0.03 ± 0.02	0.32 ± 0.09	0.51 ± 0.18
Carbon	/	1.66 ± 0.33	1.79 ± 0.25	2.53 ± 0.44

Notes: B5 = 5% biochar; B10 = 10% biochar; B15 = 15% biochar.

**Table 7:** Content of nutrients in growth medium and mortar leachates.

	Concentrations of Selected Anions in the Leachate (mg L^−1^)		
	Duckweed Growth Medium	Conventional Mortar	B5 Mortar
Nitrate (NO_3_^−^)	359.43 ± 0.02	366.07 ± 0.09	411.66 ± 0.07
Nitrite (NO_2_^−^)	0.01 ± 0.00	0.02 ± 0.00	0.00 ± 0.00
Phosphate (PO_4_^3−^)	64.68 ± 0.07	0.69 ± 0.05	0.41 ± 0.02
Sulfate (SO_3_^2−^)	48.00 ± 0.03	39.00 ± 0.00	34.00 ± 0.01
Chloride (Cl^−^)	<0.5	4.10 ± 0.02	7.20 ± 0.03

Notes. B5 = 5% biochar.

**Table 8:** Results of ICP-OES.

	Concentration of Selected Elements in the Leachate (mg L^−1^)		
	Duckweed Growth Medium	Conventional Mortar	B5 Mortar
Ca	49.6	205.7	200.3
K	141.1	112.7	128.5
S	15.0	10.8	11.2
Mg	10.4	6.7 × 10^−8^	2.6 × 10^−9^
Na	0.67	1.3	1.6
Sr	6.9 × 10^−3^	1.8	1.8
Si	3.7 × 10^−2^	0.7	0.7
Ba	0.5 × 10^−3^	272.5 × 10^−3^	277.5 × 10^−3^
Al	9.4 × 10^−3^	1.3 × 10^−2^	2.1 × 10^−2^
B	1.6 × 10^−2^	4.4 × 10^−3^	5.8 × 10^−3^
Li	<0.5 × 10^−3^	1.9 × 10^−2^	1.4 × 10^−2^
Cr	<3.5 × 10^−3^	5.9 × 10^−3^	9.4 × 10^−3^

Notes. B5 = 5% biochar.

**Table 9:** CO_2_-equivalent of Portland cement, natural aggregate (sand), and wood waste biochar.

Component	CO_2_-eq	Reference
Portland cement	1.002 kg CO_2_ kg^−1^	[62]
Natural aggregate (sand)	2.21 × 10^−3^ kg CO_2_ kg^−1^	[63]
Biochar from wood waste	−1.25 kg CO_2_ kg^−1^	[61]

**Table 10:** CO_2_-eqivalent of conventional mortar and B5 mortar mix.

Mortar Mix	Portland Cement (kg)	Sand (kg)	Wood Waste Biochar (kg)	CO_2_-eq (kg) m^−3^ of Mortar
Conventional mortar	450.0	1673.62	/	454.60
B5	427.5	1673.62	9.29	420.44

Notes. B5 = 5% biochar.

## Data Availability

The original contributions presented in this study are included in the article. Further inquiries can be directed to the corresponding author (M.L.).
